# Extracellular vesicle-based drug delivery systems for cancer treatment

**DOI:** 10.7150/thno.37097

**Published:** 2019-10-17

**Authors:** Sierra Walker, Sara Busatto, Anthony Pham, Ming Tian, Annie Suh, Kelsey Carson, Astrid Quintero, Maria Lafrence, Hanna Malik, Moises X. Santana, Joy Wolfram

**Affiliations:** 1 Department of Transplantation/Department of Physiology and Biomedical Engineering, Mayo Clinic, Jacksonville, FL, 32224, USA; 2 Department of Biology, University of North Florida, Jacksonville, FL, 32224, USA; 3 Department of Nanomedicine, Houston Methodist Research Institute, Houston, TX, 77030, USA

**Keywords:** cancer therapy, drug delivery, extracellular vesicle, nanomedicine

## Abstract

Extracellular vesicles (EVs) are naturally occurring cell-secreted nanoparticles that play important roles in many physiological and pathological processes. EVs enable intercellular communication by serving as delivery vehicles for a wide range of endogenous cargo molecules, such as RNAs, proteins, carbohydrates, and lipids. EVs have also been found to display tissue tropism mediated by surface molecules, such as integrins and glycans, making them promising for drug delivery applications. Various methods can be used to load therapeutic agents into EVs, and additional modification strategies have been employed to prolong circulation and improve targeting. This review gives an overview of EV-based drug delivery strategies in cancer therapy.

## 1. Introduction

The term extracellular vesicle (EV) refers to a wide variety of biological nanoparticles that engage in cell-to-cell communication (paracrine and autocrine) within micro and macroenvironments 1. These secreted membrane-contained nanoparticles are formed from the cell membrane (microvesicles, typically 50-1,000 nm), multivesicular bodies (exosomes, typically 40-100 nm), or apoptotic structures (apoptotic bodies, typically 800-5,000 nm) 2-4. The Minimal Information for Studies of Extracellular Vesicles guidelines proposed by the International Society for Extracellular Vesicles (ISEV) should be followed in regard to EV nomenclature. The general term EV should be used unless direct evidence of biogenesis exists (e.g. microscopy). In cases where proof of basic EV characteristics, such as size, lipid bilayer, and protein markers are lacking, the term extracellular particle should be used 2. Many cell types have been found to secrete EVs, and this is likely a universal feature of all cells due to the dynamic nature of the cell membrane. Namely, from a pure physicochemical perspective, the absence of any form of membrane blebbing during the life span of a cell is improbable. However, in most cell types EV formation is linked to a biological function and includes some type of active sorting. The biological activity of EVs depends on the surface composition and cargo, which typically consists of RNAs, proteins, lipids, carbohydrates, and in certain cases DNA 3, 5. The composition of EVs can be representative of the biological status of a cell, and usually differs from the cytoplasmic content. EVs are involved in many physiological and pathological processes, such as cancer pathogenesis. The complex process of tumor growth encompasses communication between multiple cell types, including cancer cells, endothelial cells, fibroblasts, and immune cells 6-8. EVs have been found to transfer biomolecules, such as proteins and nucleic acids, which promote tumor growth and progression within the primary organ or secondary sites of metastasis 9, 10. Despite being involved in tumor progression, EVs from both healthy and diseased cells can be exploited for therapeutic purposes as targets, immunomodulators, and drug delivery vehicles 1, 10-12. Due to the broad scope of possible EV applications, this review focuses on the use and efficacy of EVs derived from eukaryotic cells as drug delivery systems for cancer treatment. Other approaches involving EV-like particles used as anti-cancer vaccines 13 or antiviral therapeutics 13 are not reviewed.

Despite substantial contributions to cancer treatment, chemotherapy is prone to rapid clearance, poor bioavailability, low intratumoral delivery, unspecific cytotoxicity, and consequent systemic side effects, frequently followed by the onset of tumor resistance 14. To overcome these challenges, a plethora of synthetic nanodelivery vehicles have been developed, some of which are clinically approved 15, 16. In addition to reducing renal clearance and improving site-specific delivery through shape (e.g. discoidal particles to enhance interactions with tumor vasculature) 17 and/or ligand-based 18, 19 tumor tropism, artificial drug carriers enable simultaneous delivery of multiple therapeutic agents 20, 21, protection from enzymatic degradation 22, 23, immunoevasion 24, 25, sequential multistage release 26, 27, stimuli-responsive activation 17, 28, and theranostic capabilities 29. Nevertheless, the majority of these features are not yet in clinical use, partially due to complex and costly manufacturing required to achieve multi-functionality. The largest category of clinically approved nanoparticles is liposomes, which consist of a simple lipid bilayer surrounding an aqueous compartment 30. Liposomes are versatile drug delivery vehicles, as both the lipid membrane and interior space can be utilized for loading of hydrophobic and hydrophilic drugs, respectively. Similarly, EVs can be utilized as drug carriers for both water-soluble and non-water-soluble therapeutic agents. Additionally, EVs represent promising alternatives to synthetic nanoparticles, as they can exhibit intrinsic organotropic and tumor-targeting abilities 31. For instance, EV-associated integrins (ITGs) have been shown to direct tissue-specific colonization by interacting with target cells. Such ITGs bind to receptors found in the extracellular matrix such as laminin or other compatible ITGs 32. For example, ITGα6, ITGβ4, and ITGβ1 are present in lung-tropic EVs, while ITGβ5 has been found in liver-tropic EVs 32.

**Figure 1:**
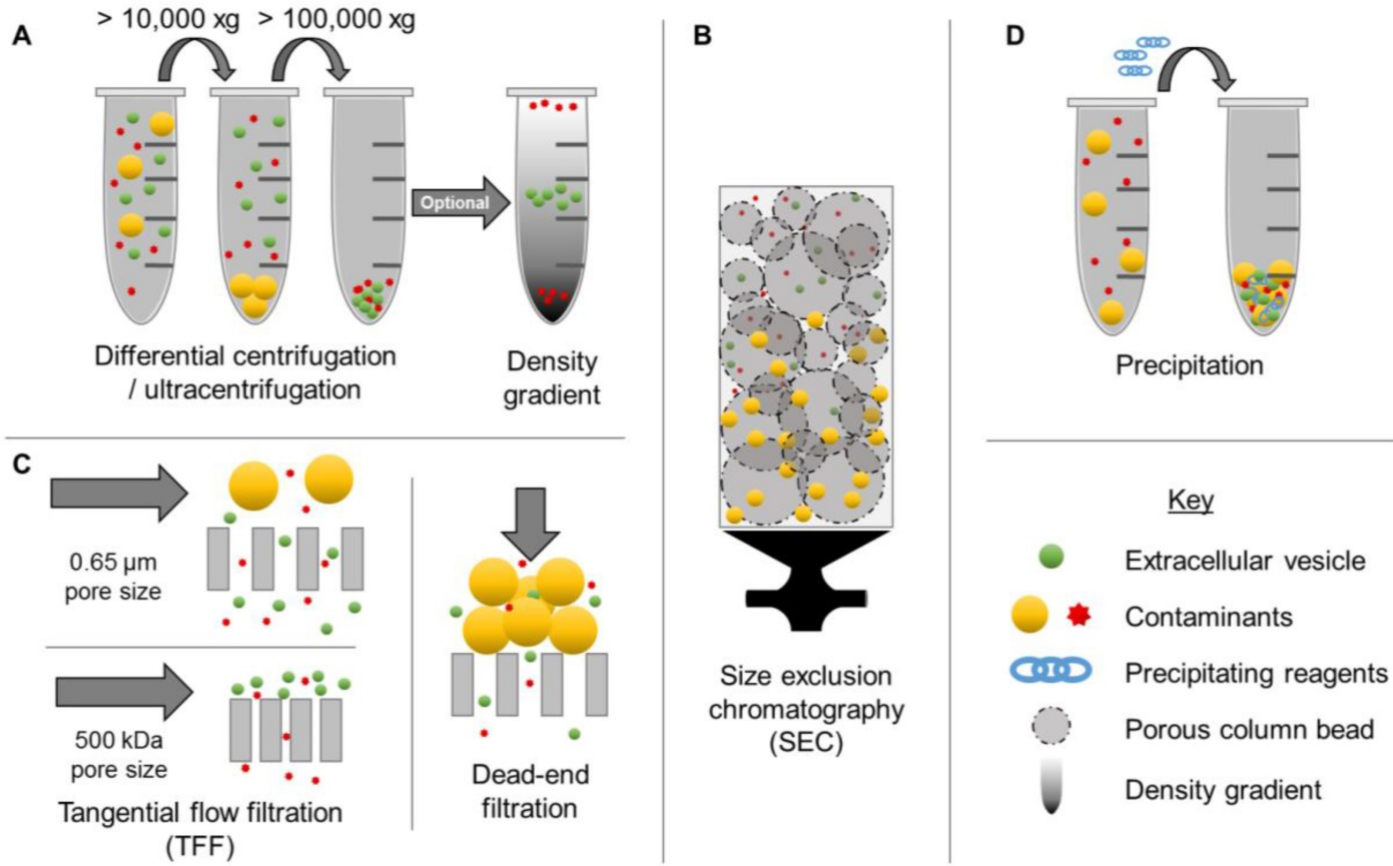
** Examples of extracellular vesicle (EV) isolation methods**. Commonly used isolation techniques include ultracentrifugation (A), differential centrifugation (A), tangential flow filtration (C), size exclusion chromatography (B), and precipitation (D). These methods result in various levels of purity.

The ability to obtain EV populations that display favorable properties could enable exploitation of complex multifaceted drug delivery mechanisms while circumventing complicated manufacturing processes. However, one of the major limitations of EV-based drug delivery has been the lack of efficient isolation methods. Although a variety of options exist for concentrating EVs from biological sources, including centrifugation (high speed, differential, and density-gradient) (Figure 1A), size exclusion chromatography (SEC) (Figure 1B), membrane affinity columns, filtration (Figure 1C), and precipitation (Figure 1D), these methods display several limitations. In particular, conventional EV isolation techniques have limited yields, low purity, and inadequate batch-to-batch consistency 2. For example, precipitation-based methods have been found to co-precipitate both larger and smaller contaminants. Although this method is capable of concentrating EVs from biological samples with low volumes, a recent study demonstrated that 9-15% of plasma proteins and 21-99% of non-EV associated microRNAs (miRNA) co-precipitate, resulting in low purity 33. Newer methods, such as tangential flow filtration represent promising alternatives for improving time-efficiency, reproducibility, and quality 34, thereby opening up new opportunities for clinical translation. Ultracentrifugation, SEC, and filtration techniques depend primarily on particle size for separation, thus nanoparticles with overlapping ranges, such as microvesicles and exosomes, are indistinguishable by these isolation methods 35. Therefore, it is also critical to thoroughly characterize EVs according to minimal criteria reported by ISEV 2. In particular, morphology, concentration, presence of EV-enriched markers, and lack of contaminant markers should be evaluated, in addition to size distribution 2.

## 2. Pharmacokinetics

Nano-sized particles are recognized by the immune system through various processes, many of which originate from defense mechanisms against bacteria and viruses, which have similar dimensions. Immunorecognition often leads to nanoparticle accumulation in the liver due to size-dependent infiltration through vascular fenestrations 36 and uptake by resident macrophages 24, which is in contrast to the rapid renal clearance seen with chemotherapy. Both synthetic 37 and biological 38 nanoparticles are prone to rapid hepatic clearance due to immunological recognition, which limits intratumoral accumulation. Although most studies have reported that EVs are cleared from circulation in a few minutes 39, 40, pharmacokinetics are likely highly dependent on EV source, subpopulation, isolation procedure, labeling method, and administration route. In fact, EV isolation and modification procedures may lead to membrane damage, which could trigger the immune system. Furthermore, it is possible that molecules used for EV tracking cause immunorecognition. As can be expected, studies have also demonstrated that the use of different administration routes result in distinct biodistribution patterns 41. For example, EVs isolated from milk were fluorescently labeled and administered intravenously or by oral gavage in mice. The mice that received intravenous injections had nearly three times as much accumulation in the liver compared to the mice that received labeled EVs orally 24 hours post-treatment 42. Distribution to other internal organs such as lung, kidney, pancreas, spleen, ovaries, colon and brain had similar patterns with no statistical differences between administration routes 42. Another study examined the effects of intravenous, intraperitoneal, and subcutaneous injections of fluorescently labeled EVs from human embryonic kidney cells. After 24 hours, intravenous injections led to significantly higher accumulation of EVs in the liver (intravenous: 60%; subcutaneous 2%; intraperitoneal: 5%) and spleen (intravenous: 12%; subcutaneous 30%; intraperitoneal: 35%) compared to other administration routes 43. Conversely, subcutaneous and intraperitoneal injections led to higher accumulation of EVs in the pancreas (intravenous: 3%; subcutaneous: 10%; intraperitoneal: 17%) and gastrointestinal tract (intravenous: 16%; subcutaneous: 41%; intraperitoneal: 36%) 43. In addition, studies have also explored intranasal administration of EVs to achieve brain accumulation 44.

An important aspect of using EVs for cancer therapy is intratumoral accumulation. A commonly proposed mechanism for nanoparticle deposition in tumors is the enhanced permeability and retention (EPR) effect, which was first described in 1986 45, 46. The EPR effect encompasses increased accumulation of nanoparticles in tumors due to fenestrations and impaired lymphatic drainage 15. Fenestrations cause tumor vasculature to be leakier than healthy blood vessels, enabling nanoparticle passage into the interstitium with each complete pass through the circulation. Therefore, nanoparticles with long circulation times are more likely to be subjected to EPR-based tumor accumulation. Additionally, impaired drainage and a dense extracellular matrix cause high retention of nanoparticles in tumor tissue following extravasation. However, it is debatable to what extent the EPR effect is present in human tumors, as considerable heterogeneity has been reported between patients 47-50, tumor types 51, and tumor regions 15, 48-50, 52-55. Furthermore, current preclinical models have limited ability to accurately represent the microenvironment, which is fundamental to the EPR effect 15.

Conventional chemotherapy does not exploit the EPR effect, as small molecules distribute easily in tissues in the absence of vascular fenestrations. In the case of EVs, that have short circulation times, the EPR effect is less likely to be a determining factor for tumor accumulation. However, long circulation times are not a necessity for tumor targeting, as there are other mechanisms that can enhance site-specific delivery. For example, if EVs display prominent preferential interactions with tumor vasculature, a few passes through the circulation could be adequate to achieve high intratumoral accumulation. It is also worth noting that the final localization of EVs in the body may not necessarily reflect that of the therapeutic cargo. It was recently demonstrated that EVs and various RNA cargo display different biodistribution patterns, suggesting that cargo re-packaging and further transportation takes place in the body 56. Certain cell types, such as Kupffer cells, could potentially act as central sorting facilities that either destroy or re-package EV cargo. In the case that this speculative hypothesis is correct, the vast majority of EVs would be unable to preferentially accumulate in organs other than the liver. In the context of EV-based drug delivery this would entail utilization of specific EV subpopulations from Kupffer cells or exploiting EV-specific properties that trigger favorable re-sorting mechanisms.

## 3. Drug loading

EVs can be loaded with various therapeutic agents, including chemotherapy and nucleic acids, such as messenger RNAs (mRNA), miRNAs, small interfering RNAs (siRNAs), and small nucleolar RNAs (snoRNAs) 57. Compared to chemotherapy, the delivery of nucleic acids into target cells is more challenging due to additional considerations, such as low intracellular uptake (attributed to negative charge and large size) and susceptible to enzymatic degradation in the blood and extracellular space 57. Although viral and non-viral cationic synthetic nanoparticles can be used for efficient RNA delivery *in vitro*, there are several safety concerns with the use of these strategies *in vivo* 58. For example, cationic lipid and polymer-based nanoparticles can cause cell shrinkage and vacuolization of the cytoplasm 59, as well as immunotoxicity 16. EVs are natural carriers of endogenous bioactive nucleic acids that can be transferred to target cells. Therefore, EVs may represent a promising drug delivery strategy for exogenous small molecule and RNA-based therapies. The following section will discuss various strategies for drug loading pre and post-EV isolation. It is worth noting that findings from drug-loading studies are difficult to generalize, as results are highly dependent on EV source, isolation technique, therapeutic agent, and loading protocol (Table 1).

### 3.1. Pre-loading

Pre-loading techniques involve incorporating cargo into cells that encapsulate the material during EV production. Cells can package both biologically produced components (proteins and nucleic acids) and synthetic compounds. For example, treating cancer cells with chemotherapeutic agents leads to the production of drug-containing EVs 60. In addition to small molecule therapeutics, pre-loading strategies have been utilized for EV-based delivery of oncolytic viruses (OVs). For example, OV-encapsulated EVs from liver cancer cells or erythrocytes were injected into mice bearing subcutaneous liver tumors 61. Treatment with OV-loaded EVs led to improved tumor growth inhibition compared to treatment with OVs, indicating that EVs shielded the viruses from immunological recognition and clearance.

Furthermore, genetic engineering of cells promotes endogenous packaging of overexpressed biomolecules 62. Pre-loading strategies can be time-consuming to set up, but provide continuous and simple production of drug-containing EVs. Additionally, the integrity of the EV membrane remains intact, as post-loading methods that typically damage the membrane are circumvented. Nevertheless, it can be difficult to control the amount of drug that is loaded into EVs, as this is dependent on several factors, such as transfection efficiency and cell viability.

### 3.2. Post-loading

**Figure 2:**
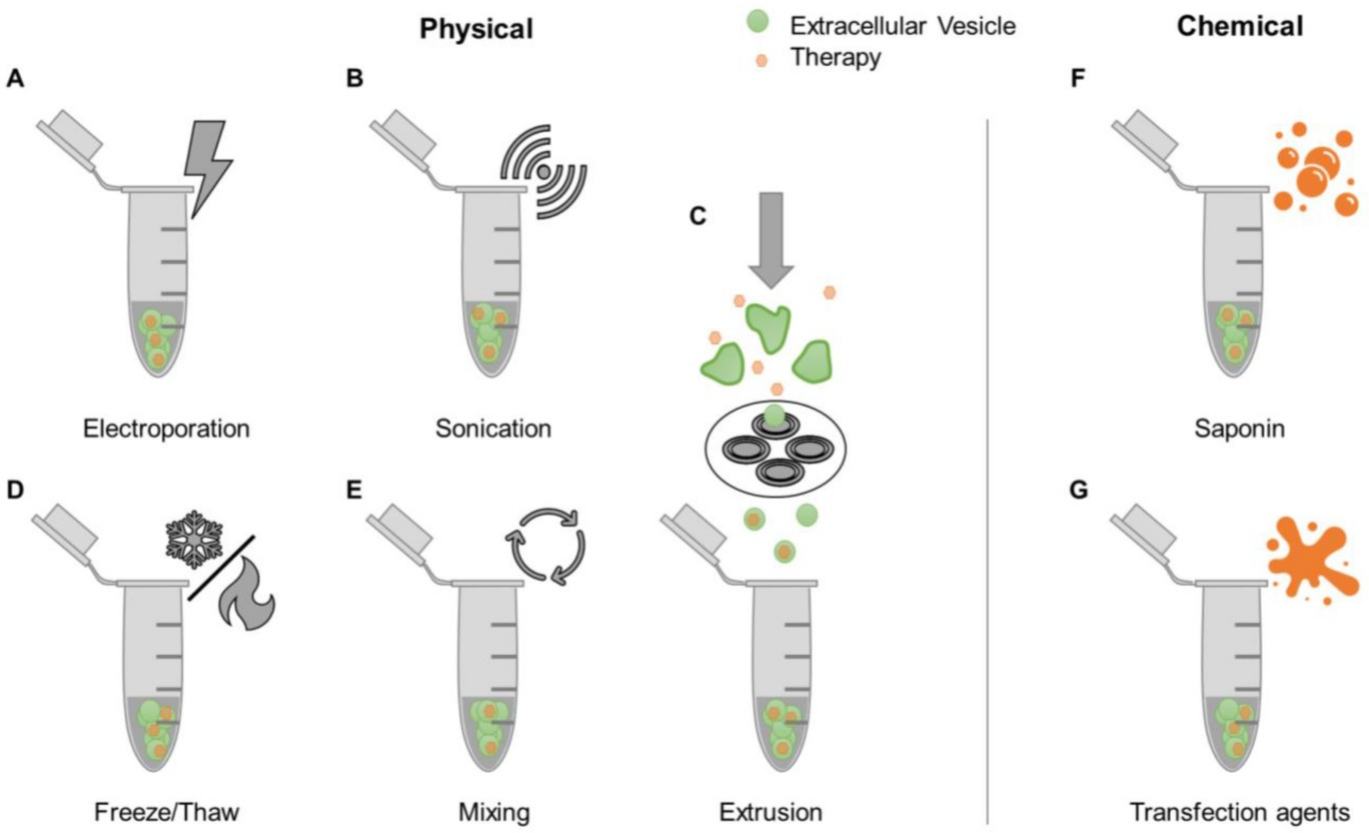
** Examples of drug loading methods post-EV isolation.** After EVs have been isolated from biological sources, drugs can be loaded into EVs through various physical [e.g. electroporation (A), sonication (B), freeze/thaw cycles (D), mixing (E), and extrusion (C)] or chemical methods [e.g. use of saponin (F) and transfection reagents (G)].

A simple strategy for loading therapeutic agents into EVs is mixing with free drugs. This approach is more efficient for hydrophobic compounds compared to hydrophilic ones 63, 64, due to the former being able to directly integrate into the EV lipid bilayer without having to cross this barrier 65. Accordingly, the hydrophobic lipid membrane presents a major obstacle for loading hydrophilic drugs into the aqueous EV interior. Various proposed mechanisms to encapsulate drugs into EVs share the common goal of bypassing the EV membrane. These methods, referred to as active loading strategies, are divided into two categories: physically-induced and chemically-induced (Figure 2). Physically-induced drug loading involves the mechanical or physical disruption of EV membranes through external forces, as seen in electroporation, sonication, freeze and thaw cycles, and extrusion. Chemically-induced drug loading uses chemical agents, such as saponin or transfection reagents, to bypass the EV membrane. The following sections will describe these methods in detail.

Electroporation involves the use of an electric field to induce spontaneous pore formation in lipid membranes (Figure 2A). The presence of the electric field disrupts the membrane, while removal of the field enables closure of pores and reformation of the lipid layer. Although electroporation typically results in low drug loading efficiencies, this method usually outperforms passive mixing (Figure 2E) when it comes to hydrophilic compounds 63. Disadvantages of this technique include the potential formation of EV aggregates and precipitated nucleic acid aggregates in RNA loading studies 64. The sonication-based drug loading method uses ultrasound energy applied through a sonicator probe that decreases the rigidity of EV membranes, enabling drug diffusion (Figure 2B). Sonication of EVs was shown to generate multiple and significant drops in membrane microviscosity, which is restored one hour post-sonication 66. Previous studies have demonstrated that small molecules, proteins, and siRNA can be loaded into EVs using this method 66-68. However, the shape and size of EVs may be affected 68. Extrusion-based drug loading is based on established protocols for formation of synthetic liposomes 69. EVs are mixed with free drugs and passed through membranes containing nanoscale pores (Figure 2C). The sheer force disrupts the lipid membrane, allowing exogenous compounds to enter EVs. The use of this method can lead to changes in EV size, composition, and delivery capacity 63, 68. The freeze-thaw cycling approach uses thermal energy to facilitate EV drug loading (Figure 2D). The formation of ice crystals temporarily disrupts the EV membrane allowing for hydrophilic compounds to enter the interior of the EV prior to membrane reconstitution due to the removal of ice crystals during the thawing cycle 70. This process has been used to load small molecules and proteins 68, 71. Freeze-thaw cycling was shown to lead to lower protein loading compared to sonication and extrusion based-methods. Similarly to the other techniques, freeze-thaw cycles may affect EV size and cause aggregates 68.

Saponin is a detergent that selectively removes membrane cholesterol, opening pores in lipid membranes (Figure 2F) 72. Saponin-treatment has been shown to be a more efficient method of EV drug loading compared to electroporation 63, 68. However, a disadvantage of saponin is potential cytotoxic effects if residues are not fully removed prior to EV use 73. Transfection agents can also be used to deliver nucleic acids into EVs by exploiting cationic substances that promote interactions with the lipid membrane and subsequent internalization (Figure 2G) 74. Lipofectamine, a well-established lipid-based transfection reagent, has been used to effectively load exogenous siRNA in EVs 75, 76. Other studies have conjugated siRNA to cholesterol moieties that associate with EV membranes through passive incubation 64, 77. The use of lipid-based agents for siRNA loading was shown to have variable effects on EV size and charge. One study demonstrated an increase in EV size attributed to siRNA-lipid conjugate integration into the EV membrane 64, while another study demonstrated consistent EV size, but decreased zeta potential that was attributed to siRNA association with EV surfaces 76. Transfection reagents should also be removed prior to EV use to avoid potential toxicity 78.

## 4. Engineering

**Figure 3:**
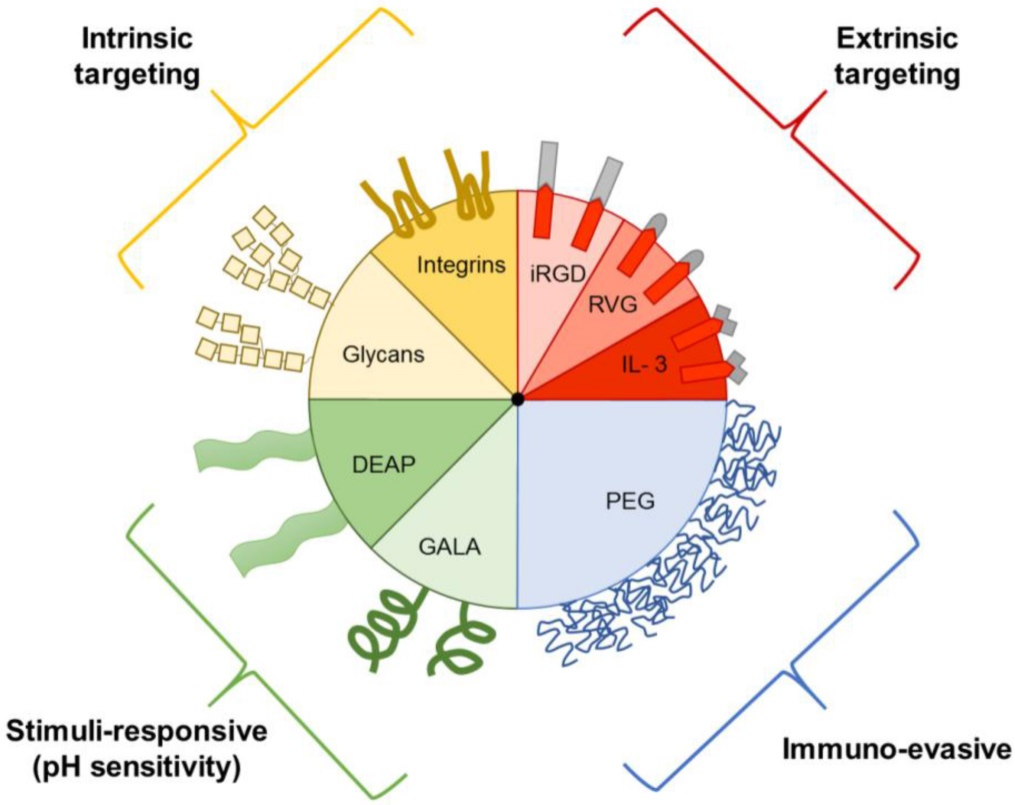
** Examples of EV components that aid in drug delivery.** EVs can express intrinsic targeting ligands, such as glycans and integrins. EVs can also be engineered to express extrinsic targeting ligands, immuno-evasive agents, and stimuli-responsive components, such as those that respond to the acidic pH of tumors. DEAP, 3-(diethylamino)propylamine; GALA, glutamic acid-alanine-leucine-alanine; IL3, interleukin 3; iRGD, internalizing arginine-glycine-aspartic acid; PEG, polyethylene glycol; RVG, rabies viral glycoprotein.

Prior to the discovery that EVs play a role in intercellular communication, they were thought to be solely involved in the elimination of waste products from cells 86. Although it is known that EVs vary in in regard to biogenesis, size, and function, the extent of EV heterogeneity exhibited by a single cell or cell line remains unknown. It is difficult to exploit innate EV transport properties for drug delivery, as isolation and characterization of specific subtypes remains challenging. Therefore, EVs have been further engineered to include targeting ligands, stimuli-responsive elements, and immune evasion properties (Figure 3) 87.

### 4.1. Targeting ligands

Altering the surface of EVs can impact biodistribution and targeting capabilities 81, 84, 88, 89. For example, surface ligands can be added to EVs through genetic engineering, wherein secreting cells are induced to express fusion proteins, or through EV post-isolation methods, such as click chemistry, a method of conjugating ligands in aqueous buffers. The following section will provide examples of strategies for incorporation of exogenous surface ligands on EVs.

EV membranes have endogenous proteins that can be fused with targeting ligands through cell engineering. This technique was first reported in 2011 in a study where dendritic cells were transfected with plasmids encoding a fusion protein for lysosome-associated membrane protein 2 (Lamp2) and the central nervous system-specific peptide rabies viral glycoprotein (RVG) 84. EVs secreted from the transfected cells were enriched with Lamp2-RVG on the external EV leaflet. After isolation, therapeutic siRNAs were loaded inside the targeted EVs using an electroporation protocol. The *in vivo* silencing of RNAs in brain regions of mice was determined following intravenous administration of targeted and non-targeted EVs 84. The results demonstrated that the targeted-EVs caused gene silencing in the brain, while the non-targeted failed to do so, indicating that the RVG peptide mediated blood-brain barrier crossing of EVs.

A similar fusion protein-based EV-engineering strategy was developed for treatment of chronic myelogenous leukemia (CML). Although the five-year survival of CML can be drastically improved with conventional therapy, i.e. tyrosine kinase inhibitors (TKIs), a subset of patients develop drug resistance and/or suffer from adverse side effects due to inefficient site-specific accumulation 88. Therefore, there is an urgent need to develop alternative therapies to improve drug delivery. CML blasts overexpress the interleukin-3 receptor (IL3-R) on the cell surface, opening up opportunities to exploit this molecule for targeting purposes. Human embryonic kidney cells were utilized as an EV source due to ease of transfection and ability to produce large amounts of EVs. The cells were transfected with a plasmid encoding a Lamp2-IL3 fusion protein and cultured in media supplemented with Imatinib (TKI). The therapeutic efficacy of IL3-engineered EVs encasing Imatinib was assessed in cell culture and mouse models. Compared to untargeted EVs, the IL3-EVs displayed improved cytotoxic effects in two CML blast cell lines, leading to decreased breakpoint cluster region-Abelson (BCR-ABL) murine leukemia viral oncogene phosphorylation in a dose dependent manner 88. Notably, the improved cytotoxicity of engineered EVs was mediated by IL3 targeting as demonstrated by a competitive binding assay. In immunodeficient mice bearing subcutaneous CML tumors, intraperitoneally injected fluorescently labeled (lipophilic dye) IL3-EVs displayed increased intratumoral accumulation compared to non-targeted EVs and free dye. Furthermore, treatment with Imatinib-loaded IL3-EVs lead to dramatically prolonged survival times and reduced tumor burden compared to non-targeted EVs and free Imatinib. In addition to Imatinib, the engineered EVs were used as a drug delivery system for BCR-ABL siRNA. IL3-EVs loaded with siRNA reduced cancer cell viability in a time and dose-dependent manner in both regular and Imatinib-resistant cell lines 88. In tumor models, the siRNA-loaded IL3-EVs also displayed efficient gene silencing, leading to delayed tumor growth.

In another study, dendritic cells overexpressing Lamp2b fused to the internalizing arginine-glycine-aspartic acid (iRGD) peptide targeting αv ITG were used to generate EVs 81. *In vitro* analysis showed that iRGD-EVs were taken up faster and to a greater extent in MDA-MB-231 breast cancer cell lines (expressing ITGαv), compared to untargeted dendritic cell EVs. Confocal microscopy demonstrated that iRGD-EVs co-localized with cancer cell membranes in a mere five minutes, while non-targeted EVs took 60 minutes to display a similar degree of co-localization. Flow cytometry demonstrated uptake efficiencies of 95.4% and 35.0% after two hours for iRGD-EVs and control EVs, respectively 81. Furthermore, when iRGD-EVs were loaded with the chemotherapeutic agent doxorubicin (Dox) through electroporation, equivalent cytotoxic effects as free Dox were observed, while drug-loaded non-targeted EVs failed to cause a reduction in cell viability 81. In an orthotopic MDA-MB-231 tumor model, Dox-loaded iRGD-EVs suppressed tumor growth, while non-targeted EVs and free Dox failed to do so 81. In Dox treatment regimens, one of the main side effects is cardiac injury. In the aforementioned *in vivo* study, reduced cardiotoxicity was observed with targeted EVs compared to non-targeted 81.

In addition to utilizing EV membrane proteins, studies have indicated that fusion proteins incorporating hydrophobic transmembrane ligands can serve to enrich targeting ligands on the cell surface 90. Cells can then be treated with surfactants to induce the formation of vesicles that express surface ligands. Compared to targeted liposomes these vesicles display improved targeting capabilities attributed to optimal orientation of proteins on the membrane surface. Accordingly, conjugation of proteins to the surface of synthetic nanoparticles can result in undesired orientations that hinder targeting. Thus, these results demonstrate the feasibility of using fusion protein-based EV engineering strategies for delivery.

Other EV-based engineering strategies for incorporation of targeting ligands include the use of post-isolation click chemistry, such as copper-catalyzed azide-alkyne cycloaddition. Click chemistry is a method by which ligands can be added to the surface of EVs without the use of solutions that damage biological components 89, 91. Accordingly, this method can be performed in aqueous buffers, and studies have shown that native properties of EVs are preserved after click-chemistry conjugation. For example, following conjugation with azide-fluor 545, EV size and cellular uptake remained unchanged 89. Intracellular delivery of protein was increased by targeting EVs via click chemistry when compared to free-protein incubation 92. Briefly, EVs were concentrated from B16F10 cells (a mouse melanoma cell line) treated with L-azidohomoalanine (AHA) and conjugated to biotin using a dibenzobicyclooctyne (DBCO)-polyethylene glycol (PEG)_4_ construct. The resulting EVs were loaded passively through incubation with streptavidin (a natural biotin ligand)-bound horseradish peroxidase (HRP). The EVs were able to deliver active streptavidin-HRP into B16F10 cells with six times higher uptake levels compared to incubation with the free protein 92. Click chemistry-based EV engineering was found to be more efficient compared to traditional cross-linking methods 62, 89.

Overall, incorporation of targeting ligands in EVs has demonstrated promising results in increasing delivery of therapeutic agents into specific cells and organs, some of which are shielded by specialized obstacles, such as the blood brain barrier. However, as targeted synthetic nanoparticles have consistently failed in the clinic 15, it remains to be seen whether EVs will face similar challenges. For example, exogenous peptides such as RVG could trigger immune responses, resulting in accelerated clearance and potential immunotoxicity. Incorporation of targeting ligands could also damage the EV structure and reduce biocompatibility. In addition to these challenges, functionalization with targeting ligands requires complex and expensive protocols that are difficult to standardize and scale up. It is also important to note that current approaches to incorporate targeting ligands on EVs require genetic (fusion proteins) or metabolic (click chemistry) engineering of cells prior to EV isolation, as traditional ligand functionalization protocols in nanomedicine are performed in conditions that would be harmful to EVs. Consequently, it would be challenging to add exogenous surface ligands to EVs that have been obtained from other sources, such as plasma or tissue samples.

### 4.2. Stimuli-responsive

Aside from adding targeting ligands to the EV membrane surface, functionality can be introduced by adding peptides that are sensitive to the environment. Tumors have an acidic extracellular microenvironment (pH 6.5 -7.2), while all cells have an acidic intracellular endosomal environment (pH 5.0-6.5) compared to the physiological pH of 7.4 93, 94. Various studies have sought to exploit these differences for EV-based drug deliver by utilizing pH-sensitive functional groups. For example, EVs have been modified with 3-(diethylamino) propylamine (DEAP), which remains associated with the lipid membrane at pH 7.4, but disrupts the membrane below pH 7.0, enabling drug release 82, 95. This pH-sensitive mechanism enables extracellular release following exposure to the acidic tumor microenvironment and intracellular release following internalization of EVs into acidic endosomes. Intracellular release is attributed to the proton sponge effect, in which protonation of DEAP draws H^+^ and Cl^-^ into the endosome contributing to osmotic swelling and rupture 94, 95. Indeed, *in vitro* studies have demonstrated enhanced drug release from DEAP-EVs at pH 6.5 compared to pH 7.4 82, 95. In HCT-116 human colorectal carcinoma tumor-bearing mice, treatment with Dox-loaded DEAP-EVs resulted in increased tumor accumulation of Dox and greater reduction in tumor volume compared to free drug and pH-insensitive Dox-loaded EVs 82.

Another mechanism for pH-sensitive intracellular drug release was demonstrated using EVs incorporating a pH-sensitive glutamic acid-alanine-leucine-alanine (GALA) peptide 96. Following endocytosis of the modified EVs into cancer cells, the GALA peptide formed helical structures in the acidic endosomal environment leading to fusion of endosomal and EV membranes as well as the release of the EV content into the cytosol. Treatment of HeLa human cervical cancer cells with these pH-sensitive EVs loaded with fluorescently-labeled dextran demonstrated GALA peptide dose-dependent delivery of EV cargo into the cell cytosol 96. Additionally, treatment of cancer cells with pH-sensitive EVs loaded with a protein synthesis inhibitor resulted in a significant reduction in cell viability compared to drug-loaded pH-insensitive EVs 96. The successful development of pH-sensitive EVs, suggest that other types of stimuli-responsive strategies could be utilized in the future.

### 4.3. Glycan stripping

Aside from adding targeting ligands to the surface of EVs, transport properties can be changed be removing endogenous surface molecules. The protein and RNA composition of EVs has been extensively studied, while the perhaps equally important glycome has been overlooked 97. Glycosyltransferases, which dictate the composition of the glycome, have previously been linked to cancer progression 98-100. In particular, glycosylation of cell surface lipids and proteins plays an important role in metastatic spread 101, and could have similar implications for EVs. The glycan sialic acid has been linked to metastatic cancers 102, and can also be found on the surface of EVs. A study demonstrated that liver progenitor cell-derived EVs exposed to neuraminidase, an enzyme that removes terminal sialic acid residues, significantly increased lung accumulation following intravenous injection in a mouse model 5. Furthermore, enzyme-treated EVs displayed a trend of increased accumulation in axillary lymph nodes following subcutaneous injection 5. These results demonstrate that alteration of the EV glycome influences biodistribution, which should be further explored for drug delivery purposes.

### 4.4. Immuno-evasive

In the context of synthetic nanoparticles, various strategies have been developed to avoid activation of the immune system, including polymer coatings that reduce interactions with cells 103, 104 and pre-treatment strategies that deactivate macrophages 105-107. Among these strategies, surface modification with polyethylene glycol (PEG), termed pegylation, is the most common approach, and several nanoparticles approved for cancer therapy incorporate this polymer 15. PEG masks nanoparticles from immune cells by forming a hydration layer, leading to reduced recognition, decreased uptake, and prolonged circulation times 108. Recent studies have evaluated the utility of PEG for EV-based drug delivery. For example, in a mouse study, EVs derived from human epidermoid carcinoma cells were fused with PEG micelles through mixing at 40°C. These EVs were detectable in circulation after one hour, while non-pegylated EVs were cleared from the blood within ten minutes 109. In addition to micelles, EVs have been fused with pegylated liposomes to prolong circulation times. For example, EVs concentrated from mouse macrophages were fused with liposomes through a freeze-thaw process to obtain hybrid properties, including the presence of PEG 70. In addition to prolonged circulation times, liposome fusion also enables highly efficient drug loading of EVs 62, 70, as well as incorporation of targeting ligands 70. In regard to EV pegylation, the accelerated blood clearance (ABC) phenomenon could be an issue, as animal studies with pegylated liposomes have demonstrated rapid antibody-mediated clearance due to PEG following repeated injections 110. However, it is unclear whether the ABC-phenomenon is clinically relevant, as high doses of pegylated liposomes were used in these animal studies.

## 5. Sources

Many cell types have been used as sources for EV-based drug delivery, including mesenchymal stem cells (MSCs), immune cells, and cancer cells 11 (Table 2). Future applications for EV drug carriers include both allogeneic and autologous applications, the latter avoiding potential immune responses 111. An additional consideration for selection of EV drug delivery systems is the growth conditions of the originating cells. A recent study demonstrated that cell membrane rigidity influences the efficiency of EV-based drug-delivery. Specifically, EVs isolated from cells grown in a soft 3D matrix displayed improved systemic drug delivery capacity in mouse models of cancer compared to EVs isolated from a standard 2D plastic extracellular environment 112. These softer nanoparticles are considered to have advantages for extravasation from the blood vessels, and penetration into the tumor, as softer membranes have a greater capacity for deformation 112. The following section will discuss the advantages and disadvantages of various EV sources.

### 5.1. MSC-derived EVs

MSCs are multipotent stem cells mainly found in bone marrow and adipose tissue. These cells are capable of differentiating into osteoblasts, chondrocytes, and adipocytes *in vitro* in response to specific growth factors 113, 114. MSC are thought to possess limited immunogenicity due to low expression of co-stimulatory molecules, such as class I major histocompatibility complex (MHC) molecules, making them suitable for allogeneic transplantation 11, 38. These cells have been found to migrate to tumors and sites of inflammation while displaying intrinsic therapeutic properties 11, 115. To date, the use of MSCs in cell therapy has been limited due to potential oncogenicity and size-based accumulation in pulmonary capillaries 38. Notably, EV secretion represents one of the main mechanisms by which MSCs exert therapeutic effects, and MSC-derived EVs inherit inflammatory tropism 38. For this reason, MSC-EVs represent an attractive cell-free strategy with lower risks that could generate comparable therapeutic effects. MSC-EVs can display therapeutic potential in cancer by modulating processes occurring in the tumor microenvironment 11, which can be further compounded by incorporation of anticancer agents in the lipid bilayer or aqueous interior. For example, MSCs transduced to express tumor necrosis factor (TNF)-related apoptosis inducing ligand (TRAIL), a membrane protein that induces cancer cell apoptosis, was used as a source of EVs 116. The cytotoxic activity of TRAIL-MSC-EVs was evaluated using a small library of tumor cell lines and compared to unmodified MSC-EVs and recombinant soluble TRAIL 116. Notably, among the cell lines tested, the non-tumorigenic human bronchial endothelial cell line was unaffected in response to any of the treatments. On the contrary, TRAIL-MSC-EVs had cytotoxic dose-dependent effects in lung and breast cancer cells, while soluble TRAIL and MSC-EVs displayed significantly lower or non-existent cytotoxic effects 116. The therapeutic mechanism of TRAIL-MSC-EVs was attributed to TRAIL receptor binding on target cells, causing activation of the caspase cascade that culminates in apoptosis. Notably, TRAIL-MSC-EVs also displayed therapeutic efficacy in TRAIL-resistant cancer cell lines 116.

### 5.2. Immune cell-derived EVs

EVs secreted from immune cells are particularly promising for cancer therapy. For example, one of the mechanisms by which natural killer (NK) cells cause cancer cell death is through secretion of EVs containing cytotoxic proteins, such as perforin (PFN), granzyme A (GzmA), GzmB, granulysin (GNLY) and Fas ligand (FasL) 11. The naive properties of NK-EVs can be further enhanced through priming cells with interleukin 15 (IL15), which causes an increase in the expression of such proteins 117. Compared to EVs released from inactivated cells, EVs secreted from cells exposed to IL15 displayed enhanced tumor targeting ability and cytotoxicity in glioblastoma, breast, and thyroid cancer cells, while the viability of kidney epithelial cells was unchanged 117. Innate tumor-targeting abilities of fluorescently labeled IL15-NK-EVs were assessed in mice bearing ectopic glioblastoma tumors and compared to that of NK-EVs and free dye 117. IL15-NK-EVs displayed longer circulating times, increased tumor-specific delivery, and prolonged intratumoral accumulation (up to 72 hours compared to 48 hours for naive EVs). Furthermore, mice treated with IL15-NK-EVs had a tumor mass that was ~30% and ~50% smaller compared to groups treated with NK-EVs and phosphate buffered saline (PBS), respectively 117.

### 5.3. Cancer-cell derived EVs

Cancer cells produce a large amount of EVs with unique homing abilities, due to microenvironmental conditions characterized by rapid metabolism and hypoxia that drive metastatic processes 10, 11, 118. Several studies have shown that cancer cell-derived EVs loaded with chemotherapy can overcome drug resistance in stem cell-like cancer cells 119, which are one of the major barriers to effective cancer treatment. Notably, in a clinical study, intrathoracic injection with cisplatin-loaded EVs isolated from A549 human lung cancer cells dramatically reduced the overall amount of cancer cells in pleural effusions, as well as the prevalence of stem cell-like cancer cells, in three end-stage lung cancer patients resistant to cisplatin 119. On the contrary, intrathoracic injection of free cisplatin failed to cause any signs of cancer regression, such as reduced pleural effusion volume, color, turbidity, and cancer cell content. The efficacy of chemotherapy-loaded EVs was also assessed in mouse models of hepatocellular and lung cancer induced through intraperitoneal or intravenous injection of cancer cells. Treatment with drug-loaded EVs decreased tumor burden and prolonged survival to a greater extent than treatment with free chemotherapy 119. The effects of cisplatin, doxorubicin, or methotrexate-loaded cancer cell-derived EVs were further explored on a small library of cancer cells 119. Notably, the EVs displayed increased anticancer activity in drug-resistant cells compared to non-resistant ones, which was attributed to enhanced uptake and intracellular retention of EVs due to characteristic cell softness and deformability in resistant cells 119. Moreover, the EVs downregulated adenosine triphosphate (ATP)-binding cassette transporter expression in resistant cells, is causing inhibition of drug efflux. The EVs were also able to shuttle therapeutic content into the nucleus of target cells through a microtubule-dependent mechanism 119. Overall, this study provides cell culture, animal, and clinical evidence that cancer-cell derived EVs are effective drug delivery vehicles. However, whether these effects are attributed to EV-specific properties or a consequence of nano-sized dimensions remains unknown. Indeed, one of the known benefits of synthetic nanodelivery is evasion of drug resistance due to endocytosis-based uptake pathways that circumvent drug release in the vicinity of efflux pumps on the cell membrane 15. Therefore, it would be important to comparatively assess the performance of chemotherapy-loaded synthetic nanoparticles, as well as EVs from non-cancerous cells. Moreover, the use of cancer cell-derived EVs for drug delivery purposes is likely to introduce endogenous cargo molecules that activate pathological pathways 9, 118, necessitating the development of methods to deactivate or remove harmful EV content. For example, it is possible that administration of cancer cell-derived EVs could promote tumor growth and metastasis of existing tumors in the body.

One of the main advantages of using cancer cell-derived EVs for therapeutic purposes is the presence of tumor-specific antigens that can prime immune cells to induce an immune response. Loading EVs with inhibitors of immunosuppressive cells or immunostimulatory compounds can further enhance therapeutic efficacy. For example, murine melanoma cells transfected with a plasmid encoding a fusion protein composed of streptavidin and the EV-enriched protein, lactadherin, enabled subsequent surface tethering with a cytosine-phosphate-guanine (CpG) DNA adjuvant linked to biotin, a natural streptavidin ligand 120. The resulting engineered EVs (CpG-EVs) were enriched in both the adjuvant and endogenous cancer cell-derived antigens, leading to improved cellular internalization and antigen-presentation by dendritic cells. Splenocytes from mice that had been intradermally injected with CpG-EVs displayed secretion of interferon-γ (IFN-γ) and cytotoxic T lymphocyte (CTL) activation upon *ex vivo* exposure to melanoma cells, while splenocytes from mice injected with CpG or a mixture of CpG and EVs did this to a much lesser extent 120. *Ex vivo* exposure of these splenocytes to a lymphocyte cell line did not result in significant IFN-γ secretion or CTL activation, indicating melanoma cell line-specific immune responses. Additionally, cancer cell line-specific humoral responses were also observed in response to CpG-EV administration in mice. CpG-EVs were capable of delaying tumor growth, reducing metastasis, and prolonging survival when administered prior to or after cancer cell inoculation, while treatment with a mixture of CpG and EVs led to negligible antitumor effects 120. Conclusively, cancer cell-derived EVs have promising potential as delivery vehicles for co-delivery of endogenous tumor antigens and exogenous adjuvants.

## 6. Comparison to other nanodelivery systems

EVs may infer advantages over synthetic nanoparticles due to intrinsic targeting capabilities, complex surface proteins, and immunoevasive properties. However, there are advantages and challenges to both modes of drug delivery. Compared to EVs, synthetic nanoparticles can be produced in a more consistent manner resulting in a homogenous population. Additionally, first and second-generation synthetic nanoparticles, some of which entered the market as early as 1995 122, have well established clinical-grade manufacturing protocols and known safety profiles. However, complex functionalization of synthetic nanoparticles for cancer therapy has consistently failed in clinical trials, especially with targeting ligands 15. One of the main reasons for this failure is protein corona-based masking of moieties on the nanoparticle surface 123. It is unclear whether EV engineering with exogenous targeting ligands would face similar challenges. Indeed, EV-based transport in the body is not well understood, partially due to a lack of studies exploring the EV-protein corona interface. Exploiting several endogenous EV surface molecules could potentially be a superior strategy for achieving organotropic targeting. Nevertheless, EVs are heterogeneous with complex molecular composition. Therefore, exploiting endogenous homing properties necessitates isolation of distinct EV subpopulations that display favorable transport properties, which has proved challenging with current techniques.

It is important to note that EV-based drug delivery studies require further side-by-side comparisons with synthetic nanoparticle controls in order to justify the use of the former. Liposomes with similar features as EV-based delivery systems (e.g. size, drug amount, drug loading method, and engineered targeting ligands) should be used for comparison purposes 124. For example, in one study synthetic fusogenic liposomes containing cholesterol-siRNA conjugates were compared to mouse melanoma-derived EVs displaying the same conjugates 125. Both carriers expressed a negative surface charge, similar size distribution, and ability to incorporate chol-siRNA. Treatment with the fusogenic liposomes caused suppression of target genes, while EVs failed to do so. Compared to liposomes EVs were taken up to a lesser extent by cells. Incubation of EVs with an endolysosomal agent led to downregulation of genes, which may indicate that the anchoring of siRNA was too stable, resulting in the cargo being trapped in the endolysosomal pathway 125. This study highlights the importance of side-by-side comparisons and illustrates that the superiority of synthetic drug carriers over EVs and vice versa may be highly context dependent (e.g. drug loading method).

Another drug delivery system that exploits natural membranes is cell membrane-coated nanoparticles (CMNPs). Comparisons with EVs should be discussed, as these carriers display similar size and surface properties. Similarly to EVs, the performance of CMNPs is highly dependent on the biological source material. For example, studies comparing erythrocyte, leukocyte, and platelet membrane coatings have noted differences in circulation time and tissue targeting 126. Specifically, red blood cell (RBC) membranes resulted in the longest NP circulation half-lives of the hematopoietic cell lines; however, RBC membranes were not found to possess innate tissue targeting capabilities, requiring further modifications 126-128. However, *in vitro* studies have demonstrated cancer-targeting capabilities of RBC-nanoparticles following modification with a targeting ligand 129, 130. Platelet membranes have been used to coat nanoparticles due to their ability to adhere to injury sites in damaged vasculature and to bind various disease-related substrates 131. These CMNPs have potential use in the treatment of cancers due to documented prevalent interactions between platelets and tumor cells 126, 132. Studies have demonstrated that platelet-nanoparticles are able to target P-selectin and CD44 receptors overexpressed on cancer cells 133.

CMNPs are being explored as alternatives to EV-based drug delivery systems due to concerns about complex and inefficient isolation methods resulting in low yields 126, 134, 135. It should be noted that CMNP isolation processes have faced similar yield limitations; a large number of cells are needed to produce enough membranes to achieve therapeutic goals 135. The benefit of CMNPs as a drug delivery system is the combined properties of synthetic nanoparticles and cell membranes. Specifically, well-established manufacturing protocols can be used to obtain homogenous synthetic nanoparticles that have been optimized to carry certain types of therapeutic agents, while the cell membrane component offers a biological solution to improve circulation times and immune tolerance of synthetic nanoparticles. A major disadvantage of CMNPs is the aforementioned issue with membrane extraction; a process that typically requires several isolation steps depending on the cell type 132, 135. Membrane orientation is also a concern, requiring modification of nanoparticles to match the charge of the inner side of the cell membrane 135, 136. Compared to cell membranes, EVs play a broader role in intercellular communication due to involvement in cellular internalization and intracellular transport. Although the intracellular faith of EVs is not well understood, future drug delivery approaches could potentially harness EV-mediated organelle targeting, which may not be a prominent feature of cell membranes. An additional benefit of EVs is the presence of endogenous cargo with possible therapeutic benefits 38, 137. In conclusion, EVs and CMNPs have distinct properties that could be beneficial; depending on the type of drug delivery application.

## 7. Conclusions

EVs offer promising prospects for improving the delivery of therapeutic agents due to intrinsic properties, such as tissue tropism. EVs have also been further engineered to maximize drug delivery potential, including incorporation of targeting ligands, stimuli-responsive components, and immunoevasive factors. Implementation of engineering strategies that have taken decades to optimize in the field of synthetic cancer nanomedicine could provide many advantages, resulting in drug delivery systems that benefit from both biological and exogenous properties.Exploiting several endogenous EV surface molecules could potentially be a superior strategyfor achieving organotropic targeting. However, this type of approach necessitates isolation of distinct EV subpopulations that display favorable transport properties, which has proved challenging with current techniques. Furthermore, EV-based transport in the body is not well understood, partially due to a lack of studies exploring the EV-protein corona interface.

In addition to limitations in separating EV subpopulations and understanding EV transport properties, scalable manufacturing remains a major hurdle for clinical translation. It is also unclear whether allogeneic EV sources, which have the potential to be streamlined and developed as off-the-shelf products, could be used for future drug delivery applications. MSC-EVs are considered particularly suitable for drug delivery applications, as they have few immunostimulatory surface markers. However, studies evaluating the allogeneic potential of EVs from various sources are lacking. Plasma transfusions rarely result in adverse immune reactions 138, although this biological fluid contains a large amount of EVs from various cells in the body 139. Therefore, it is possible that EVs from a wide variety of sources could be used for allogeneic drug delivery applications without causing immunotoxicity. However, autologous EVs may be uniquely suited for prolonged circulation and cancer immunotherapy applications.

The first clinical trials utilizing EVs were reported in 2005 140, 141, and involved subcutaneous/intradermal administration of antigen-loaded autologous dendritic cell EVs for treatment of non-small cell lung cancer and metastatic melanoma. Although these trials did not demonstrate any major therapeutic benefits, they indicated that EV therapy is clinically feasible and well tolerated. However, the lack of standardized/scalable EV isolation techniques, storage methods, and appropriate quality controls has hampered further translation and clinical-grade production of EVs. Furthermore, EV engineering approaches contribute to additional manufacturing and safety consideration that need to be addressed. An improved understanding of the abovementioned issues is likely to pave the way for the development of promising off-the-shelf or streamlined EV-based cancer therapies that would enable broader applications and commercial success.

## Figures and Tables

**Table 1:** Drug loading efficiency in EVs.

Extracellular vesicle (EV) sources	Loading content	Loading Method	Loading Measurement	Efficiency (type, %)		Ref.
Drug-based Therapy						
Raw 264.7 macrophages (mouse)	Paclitaxel (PTX)	Mixing	High performance liquid chromatography (HPLC)	Loading capacity	1.4 (SEM ± 0.38%)	66
		Electroporation			5.3 (SEM ± 0.48%)	
		Sonication			28.29 (SEM ± 1.38%)	
LNCaP and PC-3 (human)	PTX	Mixing	Ultra-performance liquid chromatography (UPLC)	Encapsulation efficiency	9.2% (SD ± 4.5%)	79
Milk (bovine)	PTX	Mixing	UPLC	Encapsulation efficiency	7.9 ± 1.0%	80
Immature dendritic cells (mouse)	Doxorubicin (Dox)	Electroporation	Fluorescence of Dox	Encapsulation efficiency	< 20%	81
Raw 264.7 macrophages (mouse)	Dox	Sonication	Fluorescence of Dox	Encapsulation efficiency	8.0-11.0%	82
Protein-based Therapy						
Raw 264.7 macrophages (mouse)	Catalase	Mixing	Catalase enzymatic activity	Loading capacity	4.9 (SEM ± 0.5%)	68
		Saponin permeabilization			18.5 (SEM ± 1.3 %)	
		Sonication			26.1 (SEM ± 1.2 %)	
		Extrusion			22.2 (SEM ± 3.1%)	
Small Nucleotide Therapies						
Plasma (human)	Mitogen activated protein kinase 1 (MAPK1) small interfering RNA (siRNA)	Electroporation	Western and northern blotting	-	Presence confirmed	83
Malignant ascites fluid (mouse)	RAD51 and RAD52 siRNA	Mixing with lipofectamine	Confocal microscopy and flow cytometry	-	Presence confirmed	76
Primary immature dendritic cells (mouse)	Glyceraldehyde 3-phosphate dehydrogenase (GAPDH) siRNA	Electroporation	qPCR analysis fluorescence microscopy	Encapsulation efficiency	10 - 38%	84
Primary dendritic cells (mouse)	Vascular endothelial growth factor (VEGF) siRNA	Electroporation	qPCR analysis	Encapsulation efficiency	3%	85

Dox: doxorubicin; GAPDH: Glyceraldehyde 3-phosphate dehydrogenase; HPLC: High performance liquid chromatography; Lamp2b: lysosome-associated membrane protein 2; LNCaP: lymph node carcinoma of the prostate; Mapk1: mitogen activated protein kinase 1; PC-3: prostate cancer; PTX: paclitaxel; UPLC: ultra-performance liquid chromatography; qPCR: quantitative polymerase chain reaction; SD: standard deviation; SEM: standard error of measurement; siRNA: silencing RNA; VEGF: vascular endothelial growth factor.

**Table 2:** Examples of EV-based drug delivery for cancer.

Extracellular vesicle (EV) source	EV isolation	Loading	Engineering (additional features)	Therapeutic cargo	Pathology^*^	Ref.
Bone marrow-derived MSCs (human)	Filtration	Cell-made (genetic engineering)	None	TRAIL	Lung cancer (*in vitro*) Pleural mesothelioma (*in vitro*) Renal cancer (*in vitro*) Breast adenocarcinoma (*in vitro*) Neuroblastoma (*in vitro*)	116
A549 lung carcinoma cells (human)	Differential gradient centrifugation	Passive incubation	None	Doxorubicin Cisplatin Methotrexate	Lung carcinoma (*in vitro*, mouse models, and stage IV human patients) Hepatocarcinoma (*in vitro*, and mouse models) Breast carcinoma (*in vitro*)	119
H22 hepatocarcinoma cells (mouse)						
Lewis lung carcinoma cells (mouse)						
MCF-7 breast carcinoma cells (human)						
ADR/MCF-7 doxorubicin resistant breast carcinoma cells (human)						
EL-4 lymphoma cells (mouse)	Sucrose gradient centrifugation	Mixing	None	Curcumin	Tumor-induced inflammation (*in vitro*, and mouse models)	65
B16-F10 melanoma cells (mouse)	Ultracentrifugation (UC)	Electroporation	None	Superparamagnetic iron oxide nano-particles	Melanoma (*in vitro*)	121
LNCaP and PC-3 prostate cancer cells (human)	Differential centrifugation	Mixing	None	Paclitaxel	Prostate cancer (*in vitro*)	79
Raw 264.7 macrophages (mouse)	Low-speed centrifugation with precipitating reagents and purifying column (ExoQuick-TC Kit, System BioSciences)	Mixing Electroporation Sonication	None	Doxorubicin and paclitaxel	Multi-drug resistant cancers (*in vitro*, and mouse models)	66
Immature dendritic cells (mouse)	Ultrafiltration, UC, and gradient centrifugation	Electroporation	iRGD-Lamp2b	Doxorubicin	Breast cancer (*in vitro*, and mouse models)	81
Milk (bovine)	Differential gradient centrifugation and UC	Mixing	None	Paclitaxel	Lung cancer (*in vitro*, and mouse models)	80
B16BL6 melanoma cells (mouse)	Filtration and differential UC	Mixing	None	CpG DNA	Melanoma (*in vitro*, and mouse models)	120
293T embryonic kidney cells (human)	Differential centrifugation and UC	Cell-made (drug treatment or genetic engineering)	IL3 - Lamp2b	Imatinib, BCR-ABL siRNA	CML (*in vitro*, and mouse models)	88
H22 hepatocarcinoma cells (mouse)	Differential centrifugation	Cell made (drug treatment)	3D-gel matrix (reduces membrane rigidity)	Doxorubicin, 5-FU	Hepatocarcinoma (*in vitro*, and mouse models)	112
B16-F10 melanoma cells (mouse)						
Raw 264.7 macrophages (mouse)	Differential centrifugation and UC	Sonication	DEAP	Doxorubicin	Colon cancer (*in vitro*, and mouse models)	82
HeLa cervical cancer cells (human)	Precipitating reagents (Total exosome isolation kit, Invitrogen)	Electroporation	GALA	Dextran	Cervical cancer *(in vitro)*	96
Primary dendritic cells (mouse)	Differential centrifugation and UC	Electroporation	Anti-nucleolin aptamer AS1411	VEGF siRNA	Breast cancer (*in vitro*, and mouse models)	85

5-FU, 5-fluorouracil; BCR-ABL, break point cluster region-Abelson; CML, chronic myeloid leukemia; CpG, cytosine-phosphate-guanine; DEAP, 3-(diethylamino)propylamine; EV, extracellular vesicle; GALA, glutamic acid-alanine-leucine-alanine; IL3, interleukin 3; iRGD, internalizing arginylglycylaspartic acid peptide; Lamp2b, lysosome-associated membrane protein 2; MSCs, mesenchymal stromal cells; siRNA: small interfering RNA; TRAIL, tumor necrosis factor-related apoptosis inducing ligand; UC, ultracentrifugation; VEGF: vascular endothelial growth factor.

## Abbreviations

5-FU: 5-fluorouracil
ABC phenomenon: accelerated blood clearance phenomenon
AHA: L-azidohomoalanine
ATP: adenosine triphosphate
BCR-ABL: break point cluster region-Abelson
CML: chronic myelogenous leukemia
CMNP: cell membrane-coated nanoparticle
CpG: cytosine-phosphate-guanine
CTL: cytotoxic T lymphocyte
DBCO: dibenzobicyclooctyne
DEAP: 3-(diethylamino)propylamine
Dox: doxorubicin
EPR effect: enhanced permeability and retention effect
EVs: extracellular vesicles
FasL: Fas ligand
GALA: synthetic peptide with a glutamic acid-alanine-leucine-alanine repeat
GzmA: granzyme A
GzmB: granzyme B
GNLY: granulysin
HRP: horseradish peroxidase
IFN-γ: interferon-γ
IL3: interleukin-3
IL3-R: interleukin-3 receptor
IL15: interleukin 15
iRGD: internalizing arginine-glycine-aspartic acid (RGD)
ISEV: international society of extracellular vesicles
ITGs: integrins
kDa: kilodalton
Lamp2: lysosome-associated membrane protein 2
mRNA: messenger RNA
miRNA: microRNA
MSCs: mesenchymal stem cells
NK: natural killer
OV: oncolytic virus
PEG: polyethylene glycol
PFN: perforin
RBC: red blood cell
RVG: rabies viral glycoprotein
siRNA: small interfering RNA
snoRNA: small nucleolar RNA
TKIs: tyrosine kinase inhibitor
TNF: tumor necrosis factor
TRAIL: TNF-related apoptosis inducing ligand
UC: ultracentrifugation
SD: standard deviation
SEC: size exclusion chromatography
SEM: standard error of measurement
TFF: tangential flow filtration
VEGF: vascular endothelial growth factor
x g: times gravity
μm: micrometer
